# Highly efficient gene editing of *Feline herpesvirus 1* using CRISPR/Cas9 combined with FACS

**DOI:** 10.3389/fcimb.2025.1660446

**Published:** 2025-08-19

**Authors:** Hai-Ming Wang, Shi-Jia Xu, Bing-Yan Cai, Wen-Ying Qiu, Hui Lu, Yan-Dong Tang

**Affiliations:** ^1^ Department of Veterinary Medicine, Jiangsu Agri-animal Husbandry Vocational College, Taizhou, Jiangsu, China; ^2^ State Key Laboratory for Animal Disease Control and Prevention, Harbin Veterinary Research Institute of Chinese Academy of Agricultural Sciences, Harbin, China; ^3^ College of Veterinary Medicine, Sichuan Agricultural University, Chengdu, China

**Keywords:** *Feline herpesvirus* 1 (FHV-1), CRISPR/Cas9, fluorescence-activated cell sorting (FACS), homologous recombination, gene knockout

## Abstract

*Feline herpesvirus 1* (*FHV-1*) is a major causative agent of feline viral rhinotracheitis and ocular lesions. Due to its large DNA genome, the construction of recombinant *FHV-1* viruses presents considerable challenges for conventional methodologies. In this study, we implemented an integrated strategy combining CRISPR/Cas9-mediated gene editing with fluorescence-activated cell sorting (FACS) to enable the rapid and efficient generation of recombinant *FHV-1* viruses. Specifically, the thymidine kinase (*tk*) gene was disrupted by inserting a monomeric Cherry (mCherry) reporter gene, and the glycoprotein E (*gE*) gene was similarly interrupted through the incorporation of a green fluorescent protein (GFP) reporter. The CRISPR/Cas9 system enables precise, site-specific genomic modifications, while FACS allows for effective enrichment and isolation of the desired recombinant viral populations. This combined approach significantly reduces the time required for recombinant virus generation from weeks to days, thereby offering substantial potential to expedite vaccine development and advance functional genomics research.

## Introduction

*Feline herpesvirus type 1* (*FHV-1*), a member of the *Alphaherpesvirinae* subfamily, is responsible for approximately 50% of diagnosed viral upper respiratory tract diseases and represents a major cause of ocular disease in domestic cats globally (Nasisse, 1990). *FHV-1* possesses a double-stranded DNA genome of approximately 134 kb, which is organized into unique long (UL) and unique short (US) regions flanked by inverted repeats (IR/TR) (Grail et al., 1991; Tai et al., 2010). Due to its large genome size and high GC content (~60%), targeted genetic modification presents considerable challenges (Tai et al., 2016). Traditional methods for generating recombinant *FHV-1* rely primarily on homologous recombination in mammalian cells. However, this approach is inefficient due to low recombination frequencies, requiring multiple rounds of plaque purification-a labor-intensive process that typically spans 3–4 weeks. An alternative strategy for manipulating the genomes of large DNA viruses involves the use of bacterial artificial chromosome (BAC)-based systems. Although BAC technology has enhanced genome editing capabilities for large DNA viruses, it still faces several limitations: (1) BAC cloning requires extensive restriction enzyme mapping and *in vitro* recombination; (2) maintaining large viral genomes in *E. coli* often leads to instability; and (3) viral reactivation following BAC transfection into mammalian cells remains inefficient (4). These constraints significantly hinder functional genomics research and the rapid development of vaccines against FHV-1.

The CRISPR/Cas9 system has revolutionized the field of genetic engineering, particularly in the context of large DNA viruses such as herpesviruses, whose complex genomes pose significant challenges to conventional homologous recombination techniques (Suenaga et al., 2014). By employing a programmable single-guide RNA (sgRNA) in combination with the Cas9 endonuclease, the CRISPR/Cas9 system enables precise double-strand breaks (DSBs) at specific genomic loci, thereby facilitating targeted insertions, deletions, or substitutions through either homology-directed repair (HDR) or non-homologous end joining (NHEJ). This method has demonstrated high efficiency in modifying herpes simplex virus (HSV) (Lin et al., 2016), and similar success has been reported for cytomegalovirus (CMV) (King and Munger, 2019), Epstein-Barr virus (EBV) (Yuen et al., 2015), pseudorabies virus (PRV) (Tang et al., 2016b; Tang et al., 2016a; Wang et al., 2023), and vaccinia virus (Yuan et al., 2015). These advancements have enabled the rapid generation of gene-deletion mutants for vaccine development and functional genomics studies. Recent studies have also shown that *FHV-1* can be genetically modified using CRISPR/Cas9, allowing for the integration of fluorescent reporter genes and other genetic elements (Tang et al., 2023; Yang et al., 2023; Yang et al., 2024). However, the current protocol requires three to seven rounds of plaque purification, which is both time-consuming and labor-intensive. This underscores the need for further improvements in homologous recombination efficiency when applying CRISPR/Cas9 to *FHV-1*. Fluorescence-activated cell sorting (FACS) offers a promising solution by enabling the high-throughput isolation of recombinant viruses expressing fluorescent markers such as GFP or mCherry. In this study, we combined CRISPR/Cas9 with FACS technology to streamline the generation and isolation of desired recombinants, reducing the experimental timeline from weeks to days.

## Materials and methods

### Cells, virus, reagent and plasmids

A feline kidney epithelial cell line (Crandell Rees feline kidney, CRFK) was cultured in Dulbecco’s modified Eagle’s medium (DMEM, Gibco, USA) supplemented with 10% fetal bovine serum (FBS, Gibco), 100 U/mL penicillin, and 100 µg/mL streptomycin at 37°C under 5% CO_2_. The *FHV-1* strain GD19, kindly provided by Professor Feng Cong, is an unpublished field isolate from a young cat suffering from severe rhinotracheitis. This virus was cultured in CRFK cells and its titer was determined by a plaque assay.

We designed two sgRNAs targeting the *FHV-1* thymidine kinase (*tk*, UL23) and glycoproteins I and E (*gI/gE*, US7-US8) genes, respectively. The targeting sites were based on our previous work. The sequences were: TK-sgRNA-1: 5’-AGACTACGCCAGTATAGCAT-3’, TK-sgRNA-2: 5’- GGCGGCGGCACATTCATCAG-3’, gI-sgRNA-1: 5’- TGGCGATTGGAACAGTTTAT-3’, gE-sgRNA-2: 5’- TAATCTGGAGGAGCGTGTAG-3’. These sgRNAs were cloned into the pX330 vector (Addgene #42330), which expresses Streptococcus pyogenes Cas9 and the respective sgRNA under the U6 promoter (Tang et al., 2016b; Tang et al., 2016a).

### Construction of homology-directed repair donor plasmids

​ For the tk region recombination​​, a donor plasmid carrying an mCherry reporter gene (pMD-18T-HR-mCherry) was constructed. The mCherry expression cassette ​​was amplified​​ using the pcDNA3.1-ERGIC vector as a PCR template (Bai et al., 2025). Simultaneously, the flanking homology arms ​​were amplified​​ using the *FHV-1* genome as a template. The primer sequences were: M-1-F: 5’-tcagatcatccccgatgtta-3’, M-1-R: 5’-TCAATAATCAATGTCcgtctgatctgtgtatgatg-3’, M-2-F: 5’-tacacagatcagacgGACATTGATTATTGACTAGT-3’, M-2-R: 5’-gcccttgctcaccatGGTGGCGCTAGCCAGCTTGG-3’, M-3-F: 5’-CTGGCTAGCGCCACCatggtgagcaagggcgagga-3’, M-3-R: 5’-gaacaccactaatgtttacttgtacagctcgtcca-3’, M-4-F: 5’-gagctgtacaagtaaacattagtggtgttccctat-3’, M-4-R: 5’-ttggtgtagtgaggtgtgac-3’. The obtained PCR products ​​were fused​​ using overlap PCR and ligated into the pMD-18T vector for sequencing.

For the *gE/gl* region recombination​​, a donor plasmid carrying a GFP reporter gene (pMD-18T-HR-GFP) was constructed. ​​GFP​​ was amplified using the pB513B vector as a template (Yang et al., 2021; Wang et al., 2022). Simultaneously, the flanking homology arms were amplified using the *FHV-1* genome as a template. The primer sequences were: G-1-F: 5’-agccggatatagacgctact-3’, G-1-R: 5’-atgtgcgctctgcccattaagtattatgctgtggt-3’, G-2-F: 5’-agcataatacttaatgggcagagcgcacatcgccc-3’, G-2-R: 5’-gaactcgtcgacgggtcaggcgaaggcgatggggg-3’, G-3-F: 5’-atcgccttcgcctgacccgtcgacgagttctagca-3’, G-3-R: 5’-caatttcgcatttatctatg-3’. The obtained PCR products ​​were fused​​ using overlap PCR and ligated into the pMD-18T vector for sequencing.

### Transfection and virus infection

CRFK cells were seeded into six-well plates. When the cell density reached approximately 70%, cells were cotransfected with 1.5 μg of pMD-18T-HR-mCherry, 1 μg of TK-sgRNA-1, and 1 μg of TK-sgRNA-2, in accordance with the manufacturer’s instructions for Lipofectamine 3000 (Thermo Fisher). At 24 hours post-transfection, the cells were infected with *FHV-1* GD19 at a multiplicity of infection (MOI) of 0.01. This procedure enabled targeted knockout of the *tk* gene and insertion of the mCherry reporter gene.

Similarly, CRFK cells were seeded into six-well plates and cotransfected with 1.5 μg of pMD-18T-HR-GFP, 1 µg of gI-sgRNA-1, and 1 µg of gE-sgRNA-2 using Lipofectamine 3000 according to the manufacturer’s protocol. At 24 hours post-transfection, the cells were infected with *FHV-1* GD19 at an MOI of 0.01. These procedures enabled the knockout of both the *gI* and *gE* genes and the insertion of the GFP reporter gene.

### Recombinant virus enrichment and sorting

Following transfection and infection, the cells were subjected to freeze-thaw cycling, then centrifuged at 5000 × g for 5 min to collect the supernatant. A small volume of the supernatant was inoculated into fresh CRFK cells and allowed to infect for 12 hours. The infected cells were subsequently resuspended in DMEM and analyzed using a BD FACS Aria III sorter (BD Biosciences) to isolate cells expressing either GFP or mCherry. The selected cells were then freeze-thawed and inoculated into new CRFK cultures. After 24–36 hours, the cells were subjected to flow cytometry again, and those expressing GFP or mCherry were sorted into 96-well plates pre-seeded with CRFK cells. Twenty-four hours after sorting, fluorescence expression was examined under a fluorescence microscope, and the data were recorded accordingly.

### Recombinant virus purification

For purification of viruses expressing either GFP or mCherry, the supernatant was inoculated onto CRFK cell monolayers in culture dishes. After incubation for 2 hours, the medium was removed, and the cells were washed twice with PBS before overlaying with 2% low-melting-point agarose. Plates were inverted and incubated at 37°C. After 72 hours, plaques were visualized under a microscope, and those expressing GFP or mCherry fluorescence were picked.

### Statistical analysis

Data represent mean ± SD of three independent experiments. Significance was determined by Student’s t-test.

## Results and discussion

**Figure 1:**
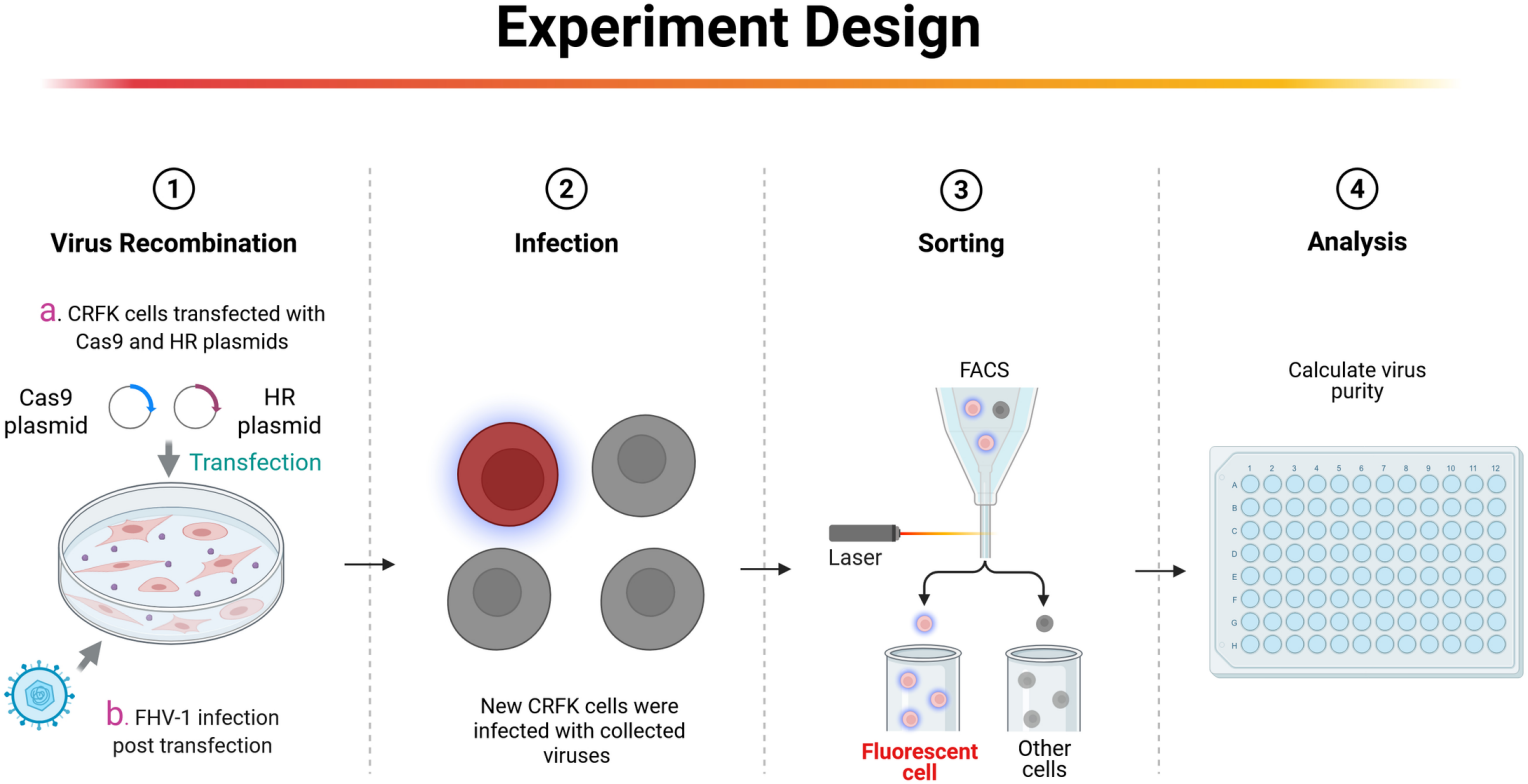
Strategy for constructing FHV-1 recombinant virus. First, CRFK cells were transfected with homology arm plasmids and CRISPR/Cas9 plasmids. After 24 hours, FHV-1 at a MOI of 0.01 is inoculated. After 48 hours of infection, the supernatant is collected and used to infect new CRFK cells. Single-cell sorting by flow cytometry is performed 12 hours post-infection to obtain the recombinant virus. This figure is created in BioRender. Li, L. (2025) https://BioRender.com/h68m2np.

**Figure 2:**
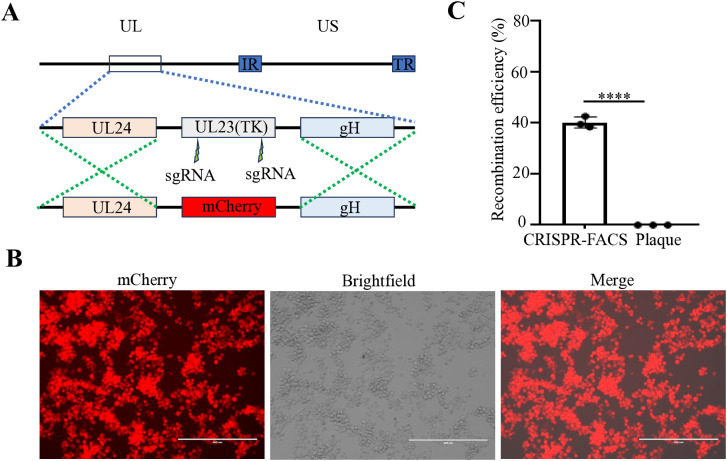
Construction of an mCherry reporter virus at the *tk* gene region of *FHV-1*. **(A)** Diagram of *FHV-1*-ΔTK-mCherry recombinant virus construction. **(B)** Fluorescence microscopy observation of a representative *FHV-1*-ΔTK-mCherry recombinant virus. **(C)** The proportion of *FHV-1*-ΔTK-mCherry recombinant virus obtained using the CRISPR/Cas9 coupled FACS method compared to the conventional method. Data represent the means ± SD from three independent experiments (An asterisk (*) indicates a significant difference between the groups (**** *p* < 0.0001).

**Figure 3:**
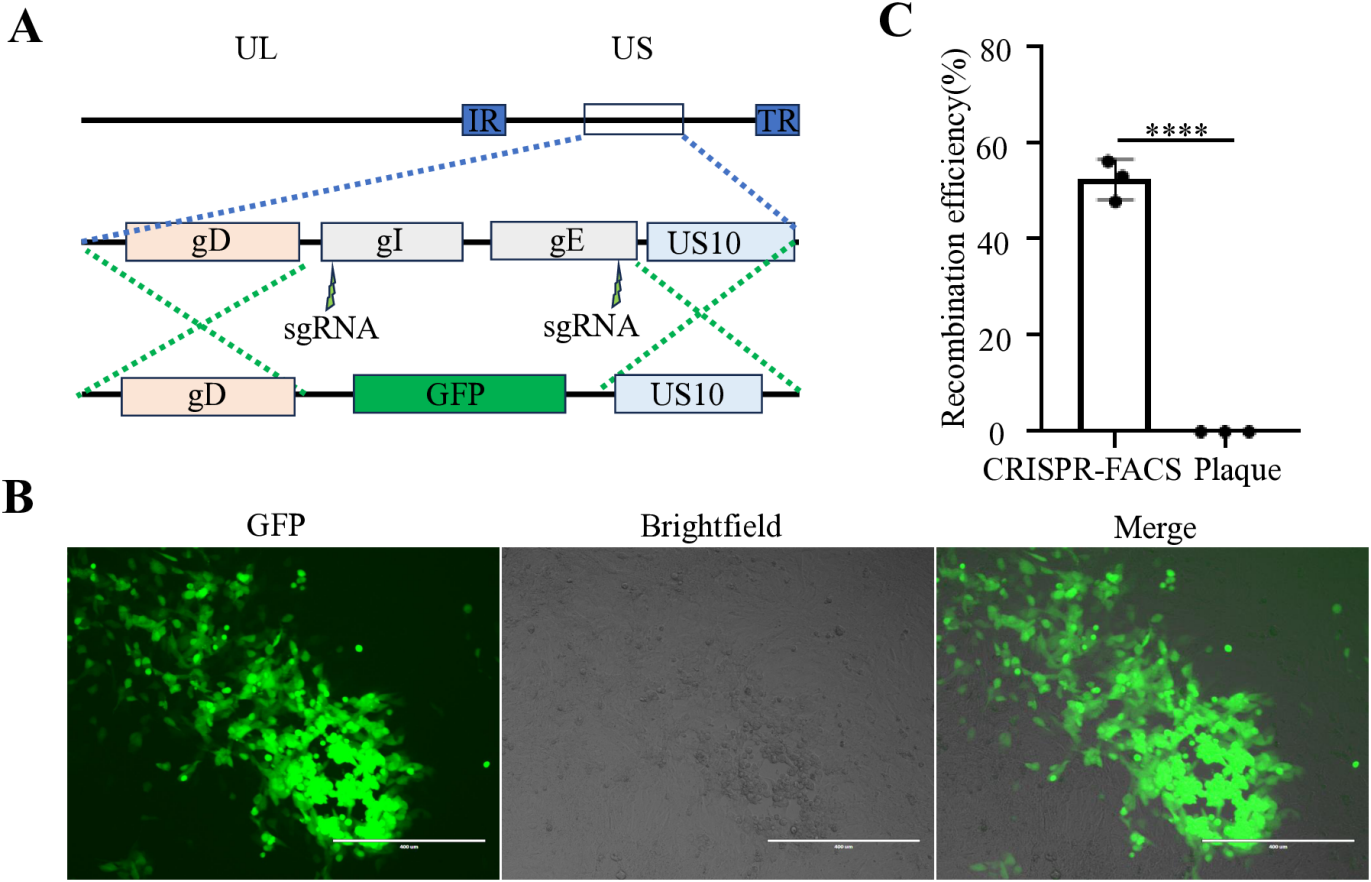
Construction of an GFP reporter virus at the *gE/gI* gene region of *FHV-1*. **(A)** Diagram of *FHV-1*-ΔgE/ΔgI-GFP recombinant virus construction. **(B)** Fluorescence microscopy observation of a representative *FHV-1*-ΔgE/ΔgI -GFP recombinant virus. **(C)** The proportion of *FHV-1*-ΔgE/ΔgI-GFP recombinant virus obtained using the CRISPR/Cas9 coupled FACS method compared to the conventional method. Data represent the means ± SD from three independent experiments (An asterisk (*) indicates a significant difference between the groups (****p < 0.0001).

Our previous studies have demonstrated that for large DNA virus manipulation, extracting the genome and employing a dual-sgRNA strategy can significantly enhance PRV genomic editing efficiency (Tang et al., 2018). Based on this approach, we extracted the *FHV-1* genome using the same PRV genome extraction protocol (Tang et al., 2018). However, transfection of the *FHV-1* genome resulted in very low virus rescue efficiency (data not shown), suggesting that the PRV genome extraction method may not be directly applicable to *FHV-1*. Subsequently, we adopted a combination of transfection and viral infection strategies to construct recombinant viruses (Tang et al., 2016b; Tang et al., 2016a; Tang et al., 2017; Wang et al., 2023; Bai et al., 2025). To improve recombination efficiency, we integrated CRISPR/Cas9 technology with fluorescence-activated cell sorting (FACS), as illustrated in **Figure 1**. Initially, CRFK cells were co-transfected with CRISPR/Cas9 plasmids and homology arm plasmids. After a 24-hour transfection period, the cells were infected with *FHV-1* at an MOI of 0.01. The virus was harvested after 48 hours and used to infect fresh CRFK cells. Twelve hours post-infection, the cells were collected and sorted by FACS. Finally, the recombination efficiency of the sorted virus was evaluated. Accordingly, we designed a knockout of the *FHV-1 tk* gene using the mCherry reporter gene, as shown in **Figure 2A**. Following FACS sorting, we successfully obtained the *FHV-1*-ΔTK-mCherry recombinant virus (**Figure 2B**). This recombinant virus was validated through plaque assays and fluorescence microscopy, revealing a recombination efficiency of 40.27% ± 1.253 (**Figure 2C**). These results indicate that the integration of CRISPR/Cas9 with FACS enables efficient construction of *FHV-1* recombinant viruses. To assess the general applicability of this method, we designed sgRNAs targeting the *gE/gl* region (**Figure 3A**) and employed the GFP reporter gene to generate recombinant viruses targeting this locus. The results confirmed successful generation of GFP-expressing recombinant viruses (**Figure 3B**), with a recombination efficiency of 52.43% ± 2.430 (**Figure 3C**).

Vaccine development plays a critical role in the prevention and control of viral diseases (Tang et al., 2024a). Several advanced technologies have contributed to the design of effective antiviral vaccines (Wang et al., 2022; Wang et al., 2023; Tang et al., 2024b). Efficient manipulation of viral genomes is essential for such vaccine development strategies. This is particularly important for large-genome viruses like *FHV-1*, which is the focus of this study. Through precise genetic modification techniques, key virulence genes can be targeted for modification, facilitating the development of more effective and safer vaccines (Tang et al., 2017). Such vaccines not only inhibit viral replication but also elicit robust immune responses. The CRISPR/Cas9 system has revolutionized genome engineering by enabling programmable, site-specific double-strand breaks via RNA-guided Cas9 nucleases. Key advantages of this system include: (1) Precision: Single-guide RNAs (sgRNAs) direct Cas9 to specific genomic loci, such as virulence genes (e.g., *tk* or *gE*), thereby promoting homology-directed repair (HDR) for the insertion of exogenous sequences. (2) Selective Enrichment: Cas9-mediated cleavage selectively suppresses wild-type virus replication, thereby enriching the population of recombinant viruses. Nevertheless, HDR rate in viral genomes remains relatively low, necessitating the implementation of effective enrichment strategies. FACS addresses this limitation by enabling high-throughput isolation of recombinant virions based on fluorescent reporters (e.g., GFP or mCherry). Specifically, FACS offers the following benefits: (1) It achieves high purity of fluorescent populations within hours, eliminating the need for time-consuming plaque assays that typically require weeks. (2) Recombinant frequencies are significantly increased compared to conventional methods. Together, the CRISPR/Cas9 system generates genetic diversity, while FACS physically isolates desired recombinants, thereby reducing the experimental timeline from weeks to days.

In our previous studies, we found that extracting the viral genome and co-transfecting it with two sgRNAs could achieve nearly complete gene knockout (Tang et al., 2018). However, when applying this strategy to *FHV-1* gene editing, the outcomes were suboptimal (data not shown). This discrepancy requires further exploration in the future. For future *FHV-1* genome manipulation, if target genes without fluorescent reporters are to be edited, they can be fused with a fluorescent reporter gene to facilitate screening during viral recombination. Once the recombinant virus is obtained, a secondary reverse selection step can be applied to remove the fluorescent reporter gene, thereby enabling rapid and efficient editing of *FHV-1*.

Overall, this platform enables the repurposing of *FHV-1* for various critical applications. Firstly, it facilitates the rapid attenuation of *FHV-1*, reducing its pathogenicity *in vivo*, making it ideal for developing live-attenuated *FHV-1* vectored vaccines. Additionally, the platform supports high-throughput analyses of viral gene functions, particularly those involved in latency. Lastly, this methodology can be applied to other large DNA viruses possessing complex genomes, such as African swine fever virus (Wang et al., 2019; Zhao et al., 2023). By addressing inefficiencies in herpesvirus engineering, the CRISPR/Cas9/FACS synergy provides a transformative toolkit that advances both fundamental virology research and translational vaccinology.

## Data Availability

The original contributions presented in the study are included in the article/supplementary material. Further inquiries can be directed to the corresponding authors.
